# Left ventricular hypertrophy: is it really?

**DOI:** 10.1186/1532-429X-15-S1-E107

**Published:** 2013-01-30

**Authors:** J Ronald Mikolich, John Lisko, Nicholas C Boniface, Brandon M Mikolich

**Affiliations:** 1 Northeast Ohio Medical University, Rootstown, OH, USA; 2 Sharon Regional Health System, Hermitage, PA, USA

## Background

Patients with left ventricular hypertrophy (LVH), typically assessed by 2D echocardiography, are at a greater risk for heart failure and sudden cardiac death. An accurate diagnosis of LVH is essential to clinical evaluation and treatment. However, 2D echo has inferior spatial resolution and less than optimal measurement positioning, relative to cardiac MRI. This "real-world" study, based on finalized 2D echo reports, sought to compare assessment of LVH on 2D echo to results by cardiac MRI (cMRI).

## Methods

All 1,255 patients in an institutional cardiac MRI database were queried for a 2D echo study within 6 months of the cMRI study. Patient 2D and cMRI reports were then classified as either negative (no evidence of LVH on the final report) or positive (evidence of LVH on the final report). Antero-septal and infero-posterior LV wall thickness dimensions were recorded for both imaging modalities for all patients, and a paired-sample t-test was used for statistical comparison.

## Results

Six hundred twenty-four patients in the cMRI database had both 2D echo and cMRI exams. Of these 624 patients, 280 patients had a diagnosis of LVH on the 2D echo report. On 2D echo the mean antero-septal LV wall thickness was 1.30 ± 0.20 cm, while on cMRI the mean antero-septal LV wall thickness was 1.20 ± 0.31 cm (p<0.001). On 2D echo the mean infero-posterior LV wall thickness was 1.29 ± 0.17 cm, while comparatively the mean infero-posterior LV wall thickness by cMRI was 0.94 ± 0.21 cm (p<0.001). Of the 280 patients with a diagnosis of LVH on the 2D echo report, the diagnosis of LVH was corroborated by cMRI in only 43 cases.

## Conclusions

There is low concordance between a diagnosis of LVH by 2D echo when compared to cMRI, as derived from final imaging reports in an active clinical practice. Because of better spatial resolution, less restricted viewing window and more accurate positioning of LV wall thickness dimensions, cMRI may be a more appropriate imaging modality for assessing left ventricular hypertrophy in a "real-world" setting.

## Funding

None. Not applicable.

